# OptoChamber: A Low-cost, Easy-to-Make, Customizable, and Multi-Chambered Electronic Device for Applying Optogenetic Stimulation to Larval *Drosophila melanogaster*

**DOI:** 10.17912/micropub.biology.001082

**Published:** 2024-04-13

**Authors:** Michael Ryan Kann, Martin K. Ackerman, Sarah D. Ackerman

**Affiliations:** 1 Department of Pathology and Immunology, Brian Immunology & Glia (BIG) Center, Washington University in St. Louis School of Medicine, St Louis, Missouri, United States; 2 University of Pittsburgh School of Medicine, Pittsburgh, Pennsylvania, United States; 3 Ackerman Tutoring, St. Louis, Missouri, United States

## Abstract

Optogenetics is a powerful tool used to manipulate physiological processes in animals through cell-specific expression of genetically modified channelrhodopsins. In *Drosophila melanogaster,* optogenetics is frequently used for temporal control of neuronal activation or silencing through light-dependent actuation of cation and anion channelrhodopsins, respectively. The high setup costs and complexity associated with commercially available optogenetic systems prevents many investigators from exploring the use of this technology. We developed a low-cost, customizable, and easy-to-make optogenetics chamber (OptoChamber) and verified its functionality in a robust cellular assay: activity-dependent remodeling of larval motor neurons in *Drosophila* embryos.

**Figure 1. The OptoChamber used to regulate dendrite volume of motor neurons in developing Drosophila melanogaster larvae:**
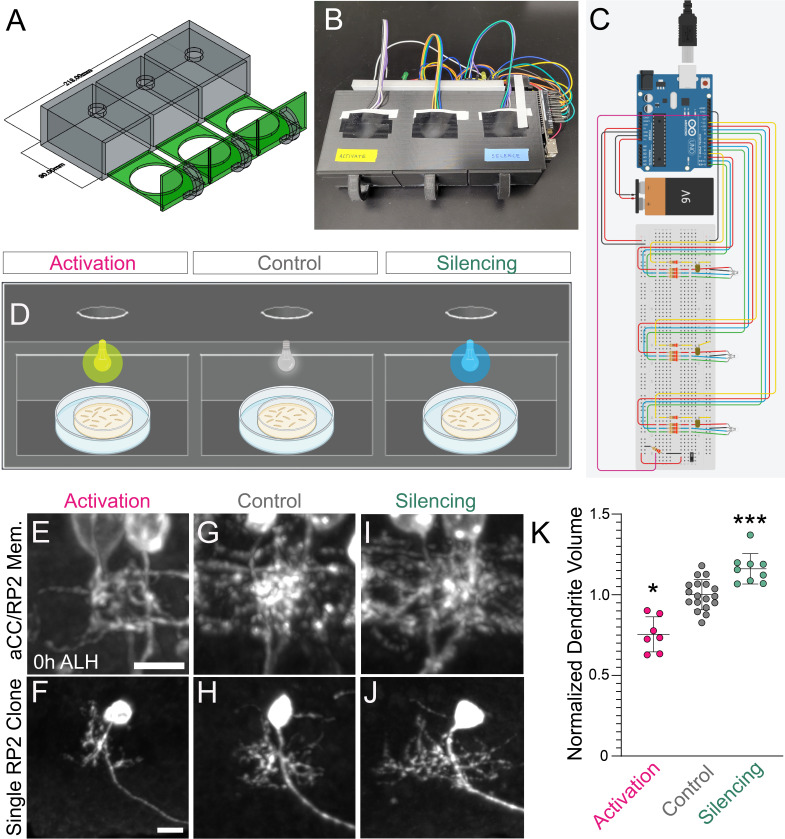
**A)** AutoCAD 3D surface reconstruction of OptoChamber. **B)** Fully constructed and functioning OptoChamber. **C)** Circuit schematic used for OptoChamber designed in Autodesk TinkerCAD. A 9V battery, or an AC to DC power adaptor, can be used as power sources. **D)** Schematic showing the experimental setup within each OptoChamber. In the activation chamber, the LED blinked at 10 Hz at a wavelength of 590nm for 1 hour (h). In the silencing chamber, the LED blinked at 10 Hz at a wavelength of 470nm for 1 h. In the control chamber, the LED produced no light for 1 hour. Figure created with Biorender.com. **E-J)** aCC–RP2 motor neuron dendrites (a single hemisegment) from dark-reared control larvae ( **G-H** ), and following activation ( **E-F** ) or optogenetic silencing ( **I-J** ) for 1 h ending at larval hatching. Full dendritic arbor (top row); RP2 clone (bottom row). Genotypes: control and silencing, *RN2-gal4,UAS-GtACR2::eYFP* ; activation, *RN2-gal4* , *UAS-CsChrimson::mCherry* ; RP2 clones, same genotypes as for silencing and activation, plus *UAS* -hsMCFO. Scale bars represent 5 µm and should be applied to all panels in a single row. **K)** Normalized dendrite volume from dark-reared control larvae (Control) and following optogenetic activation or silencing. Chrimson Controls: *n* = 6; Chrimson Activating: *n* = 7; GtACR2 Controls: *n* = 12; GtACR2 Silencing: *n* = 9. Statistics by Mann Whitney U Test: *P* < 0.05 between Chrimson Control and Activation, *P* < 0.001 between GtACR2 Controls and Silencing. Data are mean ± s.d. Biological replicates ( *n* ) from three independent experiments.

## Description

Optogenetics is a revolutionary neuromodulatory tool in which genetic sequences encoding for microbial opsins (channelrhodopsins) are expressed under the control of cell-type specific promoters, causing light sensitive channel proteins to be expressed within cells of interest (Deisseroth, 2015) . User-controlled delivery of specific wavelengths of light allows for precise spatial and temporal control of unique neuronal populations. Optogenetics was first developed nearly 20 years ago to investigate the neuronal underpinnings of behavior (Husson et al., 2013; Liewald et al., 2008) , and more recently, this technology has been applied to non-neural cells (Drozdowski et al., 2023; Jansen et al., 2015; Vogt et al., 2021) . Optogenetic tools continue to increase in popularity, and they have been applied across a variety of model organisms ranging from *C. elegans* to mice (Asakawa et al., 2021; Cermak et al., 2020; Wang et al., 2021) . *D. melanogaster,* in particular, is a perfect model organism for the application of optogenetic technology because its nervous system is well-characterized at the cellular level and there are many drivers for neuron subtype-specific manipulation (Kohsaka & Nose, 2021) .

Since the introduction of optogenetics, various experimental rigs have been created to explore the nervous system of model organisms. These systems range drastically in both their cost and functionality. The more expensive systems include custom built hardware, high powered microscopes, high speed cameras, and lasers to provide cellular resolution optogenetic control within moving organisms (Leifer et al., 2011; Stirman et al., 2011) . These systems have significant advantages by allowing stimulation of single neurons at specific anatomical positions with simultaneous tracking of behavioral output. They also often provide the ability to adjust light intensity over both short-term (minutes) and long-term (weeks) optogenetic experiments. Nevertheless, these systems are often highly customized to individual experiments and require significant setup costs, limiting their widespread use. In addition, they are frequently designed for experimentation on single animals; thus, they are low throughput.

To address this, over the past several years, multiple reports described lower-cost optogenetic systems that are accessible to a wider audience. Most notable are the OptoGenBox, the OptoArm, and the application of smartphone optogenetics (Busack et al., 2020; Koopman et al., 2021; Meloni et al., 2020) . The OptoGenBox provides adjustable temperature-controlled chambers, user-interactive touch screens, and the ability to run up to 13 individual cells that can be separately programmed for parallel optogenetic experimentation. However, at a cost of $3500, this system still poses a financial barrier for new investigators. The OptoArm describes a ~$128 dollar device that utilizes an Arduino controlled and customizable LED platform placed on the end of a long metal arm, resulting in a compact device easy to move and integrate within different recording systems. However, the device does not provide internal isolation, allowing for external light to potentially influence experimental results. Likewise, it only allows for experiments to be conducted on one petri dish at a time, limiting experimental efficiency. Lastly, the open-source smartphone app used in smartphone optogenetics achieves high spatial and temporal stimulation by taking advantage of smartphone displays to allow for the time-dependent control of light patterns across the entire color spectrum. Nevertheless, similar to the OptoArm, this device does not provide an isolated chamber and only allows for experiments to be conducted on one petri dish at a time. Additionally, while the app may be free, smartphones themselves still pose a significant upfront cost. To address the remaining limitations of previously described optogenetic systems, we describe here the production and validation of the OptoChamber: an interactive, customizable, and low-cost electronic device that can be used to apply optogenetic stimulation of larval animals.

The OptoChamber is an innovative, low-cost device that takes advantage of easy-to-use and widely available 3D printing software, basic electrical equipment (Arduino Uno, resistor, electrical wires, and a switch), and open-source Arduino coding platforms to create an interactive rig that can apply optogenetic stimulation to larval organisms. The device was designed in AutoCAD and printed with PLA filament on a standard 3D printer. It consists of a central box with three isolated chambers as well as three individual drawers, each with a central hole sized to snuggly fit a 60 mm petri dish ( Figure 1A -B). The drawers slide comfortably into the three chambers of the central box and prevent entry of external light. On top and central to each chamber is an 18 mm diameter hole through which an anode RGB LED is vertically placed. These holes are covered with black electrical tape so that when the drawers are in place, no external light can enter any of the three chambers. Likewise, the light produced from the RGB LEDs within each chamber does not interfere with the light produced in the other two chambers. The RGB LEDs are connected to a breadboard attached to the back of the central box. The electrical circuit of the device ( Figure 1C ) is programmed through an Arduino Uno, attached to the side of the central box, and powered by an AC to DC adaptor power supply, or a 9V battery ( Figure 1B ). A manual switch is also placed on the breadboard to allow users to manually override the system to turn the LEDs on/off at user-defined timepoints. Lastly, standard colored LEDs are placed on the outside of the box and are programmed to blink in unison with the internal RGB LEDs. These LEDs act as a highly visible quality check for users to ensure the internal, invisible RGB LEDs are working properly.

While standard machine shops have quoted the price of making such a device as high as $400, the total cost to create our device from scratch is <$60. This price decreases further with each unit made as the materials become cheaper when purchased in bulk. A complete materials list, including cost and supplier, is shown in Table 1. This low price point makes the OptoChamber an ideal device for teaching laboratories as well as researchers interested in exploring the use of optogenetic stimulation in larval organisms without undertaking high up-front costs. Additionally, the design of the OptoChamber creates various experimental benefits. By using a flexible AC to DC adaptor power supply, and being made with small, compact, light, and durable plastic, the OptoChamber is extremely robust and mobile. This allows it to be easily moved and placed within incubators set to different biologic temperatures. Even for cases in which there are no electric outlets within a desired location, an internal standard 9V battery attached to the back of the central box can be used in place of the AC to DC adaptor power supply to power the OptoChamber ( Figure 1B ). Note, the AC to DC adaptor is better for long-term manipulations. Depending on the battery life of the power source used, researchers can conduct stimulation experiments lasting from hours using a standard 9V battery to days using an AC to DC adaptor power supply for longer length experiments requiring a sustained power source. Likewise, by utilizing Arduino open-source coding software to program the electric circuit, the OptoChamber allows the user to adapt and customize the light frequency and wavelength of the blinking RGB LEDs as well as the total experimental time within each of the three chambers. This invention gives researchers the flexibility to adapt the OptoChamber to the context of their specific scientific question, allowing them to customize the light produced along the entire color spectrum to optimally stimulate genetically inserted light-sensitive actuators. Additionally, researchers can adjust the blinking frequency of each RGB LED to match the natural firing frequency of their target cells, mimicking physiological conditions within the animal that can lead to more physiologically relevant findings. Lastly, the barriers placed between the three isolated chambers allows multiple experiments carried out at different wavelengths, frequencies, experimental times, or even in different organisms to be performed simultaneously, which drastically increases experimental efficiency.

To test the functionality of the OptoChamber, we reproduced a previously characterized phenomenon in which activity is used to regulate dendrite length and complexity of motor neurons in developing fly larvae (Ackerman et al., 2021) . The anion channelrhodopsin GtACR2 (Mauss et al., 2017) and the channelrhodopsin Chrimson (Carreira-Rosario et al., 2018) were separately expressed within aCC-RP2 motor neurons (Landgraf et al., 2003; Tripodi et al., 2008) using the Gal4-UAS binary expression system. Chrimson is optimally activated by light at 590 nm (Klapoetke et al., 2014) , GtACR2 is optimally activated by light at 470nm (Govorunova et al., 2015) , and light pulses were delivered at 10 Hz, which is within the physiological range of aCC-RP2 motor neurons (Hunter et al., 2023) . The OptoChamber was used to provide 1-hour of activation or silencing to aCC–RP2 motor neurons in *Drosophila* larva from embryonic stage 17 through 0 hours after larval hatching (h ALH) using 590 nm or 470 nm light, respectively. Each time, the LEDs were programmed to blink at 10 Hz. The experiment was conducted using two OptoChambers. In each OptoChamber, we programmed the first chamber for motor neuron activation, the second for dark-reared control, and the third for motor neuron silencing ( Figure 1d ). Silencing resulted in a significant increase in motor neuron dendrite volume assessed at 0 hours after larval hatching (h ALH), while activation resulted in a significant loss in motor neuron dendrite volume assessed at 0 h ALH when compared to the dark-reared control ( Figure 1e -f), consistent with our previous study (Ackerman et al., 2021) . The reproducibility of these previously characterized results allowed us to confirm the OptoChamber’s ability to conduct parallel optogenetic stimulation experiments under different experimental conditions. The OptoChamber is an inexpensive, easy-to-make, easy-to-use, and highly customizable device ideal for teaching laboratories and researchers interested in exploring the use of optogenetic stimulation. Additionally, while we designed and validated the OptoChamber using *Drosophila* larva, the use cases of this device could be easily expanded to perform similar optogenetic stimulation experiments in other larval systems such as *C. elegans* and larval zebrafish.

## Methods

**OptoChamber Design and Creation**

The OptoChamber skeleton (central box, three drawers, and three drawer handles) was designed in AutoCAD 2023.1 (Product Version T.133.M.246), and it was printed with PLA filament at 20% infill and 0.2mm layer height using an Original Prusa i3 MK3(S/S+) 3D Printer. For researchers who do not have access to 3D printing services at their respective institutions, several online, reliable, and affordable 3D printing services are available such as Shapeways, Sculpteo, i.materialise, and Protolabs, among others. The circuit schematic was designed in Autodesk TinkerCAD, and the Arduino Uno was programmed using Arduino Web Editor. All STL files needed to create the OptoChamber’s central box, three drawers, and three drawer handles as well as all circuit diagrams and example code for Arduino programming can be found on our GitHub (Kann et al., 2023) . All materials used in the creation of the OptoChamber are seen in Table 1 and Table 2.

**Table 1: Materials List**

**Table:** 

**Item**	**Part Number**	**Supplier**	**Unit Price**	**Quantity**	**Link**
Solderless Breadboard Terminal Strip (No Frame)	DKS-BBOARD6.5-ND	Digi-Key	$7.99	1	Link
6.50" x 2.14" (165.1mm x 54.4mm) Red, Green, Blue (RGB) 624nm Red, 525nm Green, 470nm Blue LED Indication - Discrete 2V Red, 3.4V Green, 3.4V Blue Radial - 4 Leads	1830-1014-ND	Digi-Key	$1.02	3	Link
Jumper Wire Male to Female 6.00" (152.40mm)	1568-1511-ND	Digi-Key	$2.10	1	Link
Jumper Wire Male to Male Various	1738-1374-ND	Digi-Key	$4.70	1	Link
Chanzon SPDT Mini Micro Slide Switch 1p2t 2 Position 70pcs 3mm Toggle Vertical Slide Switch Panel Mount Small High Knob Vertical for PCB Arduino Breadboard Electronic Board Dip Miniature SlideSwitch	RA12-SLIDE-SWITCH	Amazon	$0.10	1	Link
ELEGOO UNO R3 Board ATmega328P with USB Cable (Arduino-Compatible) for Arduino	EL-CB-001	Amazon	$17.99	1	Link
EDGELEC 100pcs 220 ohm Resistor 1/4w (0.25 Watt) ±1% Tolerance Metal Film Fixed Resistor	EFR-W0P25-A:MF	Amazon	$0.55	13	Link
Wapodeai 3PCS Electrical Tape, Flame Retardant Indoor Outdoor High Temperature Resistance Electric Tape, Premium Black Waterproof Tape, 0.62 in X 49 ft	N/A	Amazon	1	$1.90	Link
LED Lighting Color HL3P Red 627nm Radial (Any color works)	516-HL3P-NR45-J00DDCT-ND	Digi-Key	3	$0.59	Link
3D Printed Box with PLA Filament	N/A	N/A	$4.31	1	N/A
3D Printed Drawers with PLA Filament	N/A	N/A	$0.42	3	N/A
3D Printed Drawer Handles with PLA Filament	N/A	N/A	$0.13	3	N/A
AC to DC Adaptor Power Supply	N/A	Amazon	$5.49	1	Link
Optional: Battery Holder (Open) 9V 1 Cell Wire Leads - 5.91" (150mm)	2368-23-BH9-6-ND	Digi-Key	$0.92	1	Link
Optional: 9V Alkaline Manganese Dioxide 9 V Battery Non-Rechargeable	3046-9V-MN1604-ND	Digi-Key	$2.46	1	Link
**Total Cost for One Optogenetics Chamber**	**$58.21 ($61.59 including optional purchase)**				

**Table 2: Recommended Bulk Sets**

**Table:** 

**Item**	**Part Number**	**Supplier**	**Unit Price**	**Quantity**	**Link**
ELOOGAA 3Pcs 830 tie Points breadboards,120Pcs 20cm (7.9inch) Dupont Cables,65Pcs Flexible Breadboard Jumper Wires,560Pcs U-Shape Jumper Wires Arduino Project kit	El24#	Amazon	$21.99	1	Link
ELEGOO UNO Project Basic Starter Kit with Tutorial and UNO R3 Compatible with Arduino IDE	EL-KIT-004	Amazon	$26.99	1	Link

***Drosophila* Husbandry and Stocks**

All flies were raised at 25 °C on standard cornmeal fly food. Animals were staged relative to a 25 °C standard. At 25 °C, embryos take 21 h to hatch into larvae (Crisp et al., 2008) . Fly stocks used in this study include *RN2-gal4* (Landgraf et al., 2003) , *20×UAS- CsChrimson::mCherry* (Carreira-Rosario et al., 2018) , *UAS* - *GtACR2::eYFP* (Mauss et al., 2017) , *10×UAS(FRT.stop)myr::smGdP-V5, 10×UAS(FRT.stop)myr::smGdP-Flag* (hsMCFO; BDSC# 64085) (Nern et al., 2015) .

**Animal Collections**

For collection of *Drosophila* embryos and staging for optogenetics, crosses were reared at 25 °C in collection bottles fitted with 3.0% agar apple juice caps containing yeast paste that was supplemented with 0.5 mM all- *trans* retinal (ATR) (Sigma-Aldrich, R2500-100MG). Crosses were supplied fresh yeast paste (+ATR) for a minimum of 72 h before embryo collection to ensure maternal transfer of ATR into embryos. We then collected embryos on 3.0% agar apple juice caps with yeast paste (+ATR) for 1.5 h and aged at 25 °C. To prevent premature optogenetic activation, crosses and embryos were reared in the dark until the appropriate developmental stage. At 25 °C, *Drosophila* embryos hatch 21 h after egg laying (AEL). For assessment of remodeling at 0 h ALH, embryos were aged to 20 h AEL before light activation or silencing in an OptoChamber for 1 h. All animals were dissected immediately following activity manipulation. Dark-reared controls were dissected under minimal light conditions (<100 lx) as done previously (Ackerman et al., 2021) .

**MultiColor FlpOut clone generation**

Embryos were prepared for heatshock to induce FLP-out clones at 6 h AEL. Apple caps covered in embryos were sliced to a thickness of ~2 mm and then adhered to a 60 mm petri dish with water to increase heat transfer. The petri dish was sealed with parafilm and floated on a 37°C water bath for 5 minutes to induce FLP-out events. Embryos were then transferred back to 25°C until the designated stage and/or manipulation.

**Optogenetic stimulation in OptoChamber**

The following strategy was used for optogenetic stimulation within the OptoChamber. All crosses and resulting embryos or larvae were maintained in dark-rearing conditions until the manipulation. Animals were maintained on 3.0% agar apple juice caps containing minimal yeast paste that was supplemented with 0.5 mM ATR. The caps with embryos aged 20 h AEL were placed within the center of a 60 mm petri dish, fit into a drawer of the OptoChamber, and slid into one of the chambers. The OptoChamber was placed in a 25 °C incubator. Caps were placed under the light path produced by the RGB LED within the chamber. Chrimson (Klapoetke et al., 2014) is maximally activated at 590 nm and minimally activated with wavelengths ≤500 nm, and GtACR2 (Govorunova et al., 2015) is maximally activated at 470 nm and minimally activated at wavelengths ≥500 nm. The OptoChamber was programmed so that an RGB LED in one chamber produced light at 590 nm at a frequency of 10 Hz for one hour (activation), an RGB LED in a second chamber produced light at 470 nm at a frequency of 10 Hz for one hour (silencing), and an RGB LED in the final chamber produced no light for one hour (dark-reared control). The millicandela rating of each RGB LED used was 600mcd red, 1000mcd green, 500mcd blue. Animals were dissected in low-light conditions (<100 lx) immediately following activity manipulations. Provision of a thin layer of ATR-supplemented yeast paste motivated larvae to remain on the top of the agar plate throughout the duration of the manipulation. Any larvae found outside of this area were not processed further.

**Immunohistochemistry**

Larval brains were dissected in sterile-filtered, ice-cold 1× PBS and mounted on 12 mm #1 thickness poly-d-lysine coated round coverslips (Fisher Scientific, GG-12-PDL). Brains were fixed for 12 min in fresh 4% paraformaldehyde (Electron Microscopy Sciences, 15710) in 1× PBS supplemented with 0.3% Triton detergent (0.3% PBST). Brains were then washed in 0.3% PBST to remove fixative. Samples were blocked overnight at 4 °C in 0.3% PBST supplemented with 1% BSA (Fisher, BP1600-100), 1% normal donkey serum and 1% normal goat serum (Jackson ImmunoResearch Laboratories 017-000-121 and 005-000-121). Brains were then incubated in primary antibody for 1 day at 4 °C. The primary antibody was removed and brains were washed overnight at 4 °C with 0.3% PBST. Brains were then incubated in secondary antibody overnight at 4 °C. The secondary antibody was removed, and brains transferred to 0.3% PBST overnight before mounting in DPX. To DPX mount, brains were dehydrated with an ethanol series: 30%, 50%, 70% and 90%, each for 5 min, then twice in 100% ethanol for 10 min each (Decon Labs, 2716GEA). Finally, samples were incubated in xylenes (Fisher Chemical, X5-1) for 2 × 10 min, were mounted onto slides containing DPX mountant (Millipore Sigma, 06552), and cured for 1–2 days before imaging.

Primary antibodies: Mouse anti-Cherry (1:500; Takara 632543); chicken anti-GFP (1:1,000; Aves GFP-1010); rabbit anti-V5 (1:500; Cell Signaling Technology 13202); rat anti-HA (1:100; Millipore Sigma 11867423001)

All secondary antibodies were purchased from Jackson ImmunoResearch and used at a working concentration of 1:400. The following antibodies were used: Alexa Fluor Rhodamine Red-X Donkey-Anti Mouse (715-295-151), Alexa Fluor 488 Donkey anti-Chicken (703-545-155), Alexa Fluor Rhodamine Red-X Donkey-Anti Rat (712-295-153), Alexa Fluor 488 Donkey-Anti Rat (712-545-153), Alexa Fluor 647 Donkey-Anti Rabbit (711-605-152).

**Light microscopy**

Fixed larval preparations for dendrite morphology analysis were imaged with a 3i spinning disc confocal using a 63×/1.4 NA Oil Plan-Apochromat DIC m27 objective lens.

**Image processing and analyses**

Quantitative analyses were performed using Imaris 10.0.0 (Bitplane).

**Figure preparation**

Images in figures were prepared as 3D projections in Imaris 10.0.0 (Bitplane) and assembled using Adobe Illustrator.

**Quantification and statistical analysis**

**Volumetric assays.** For quantification of dendrite volume, data were acquired with a voxel size of 0.169 × 0.169 × 0.27 μm. aCC–RP2 dendrites within a single abdominal hemisegment (A1–A3) were captured in a standard region of interest (ROI) spanning 100 pixels × 100 pixels in *xy* and 7 μm in *z* with the top of the ROI beginning dorsally at the aCC– RP2 axons at 0 h ALH. Dendrites were then reconstructed using the Imaris Surface module using default thresholding to determine the total dendritic volume within the ROI. Dendrite volume per brain was determined by averaging the dendrite volume of four individual hemisegments. Chrimson and GtACR2 data were normalized to time-matched, experiment-matched, dark-reared controls.

**Statistics and reproducibility.** All experiments were performed with three technical replicates with similar results. Statistics were performed using Prism (GraphPad) software. Sample sizes followed published standards. A Mann Whitney U-Test was used. Data are shown as mean ± s.d.. A 95% confidence interval was used to define the level of significance. * *P* < 0.05, ** *P* < 0.01, *** *P* < 0.001, **** *P* < 0.0001. All pertinent information, including sample size, statistical test used, and variance is seen in the figure legend or labelled within the figure.

## Extended Data

Description: Code Performance Review. Resource Type: Text. DOI: 10.22002/ppxcw-x3174

Description: Optogenetics Chamber Github Repository. Resource Type: Software. DOI: 10.22002/98ar6-d3v87
